# NHC in Imidazolium Acetate Ionic Liquids: Actual or Potential Presence?

**DOI:** 10.3389/fchem.2018.00355

**Published:** 2018-08-28

**Authors:** Isabella Chiarotto, Leonardo Mattiello, Fabiana Pandolfi, Daniele Rocco, Marta Feroci

**Affiliations:** Dipartimento di Scienze di Base e Applicate per l'Ingegneria, Sapienza University of Rome, Rome, Italy

**Keywords:** N-heterocyclic carbene, imidazolium acetate, NHC, basic anion, ionic liquids, C2-H deprotonation, organocatalytic ionic liquid

## Abstract

Ionic liquids (ILs) are considered in the majority of cases green solvents, due to their virtually null vapor pressure and to the easiness in recycling them. In particular, imidazolium ILs are widely used in many fields of Chemistry, as solvents or precursors of N-heterocyclic carbenes (NHCs). The latter are easily obtained by deprotonation of the C2-H, usually using strong bases or cathodic reduction. Nevertheless, it is known that weaker bases (e.g., triethylamine) are able to promote C2-H/D exchange. From this perspective, the possibility of deprotonating C2-H group of an imidazolium cation by means of a basic counter-ion was seriously considered and led to the synthesis of imidazolium ILs spontaneously containing NHCs. The most famous of this class of ILs are N,N'-disubstituted imidazolium acetates. Due to the particular reactivity of this kind of ILs, they were appointed as “organocatalytic ionic liquids” or “proto-carbenes.” Many papers report the use of these imidazolium acetates in organocatalytic reactions (i. e., catalyzed by NHC) or in stoichiometric NHC reactions (e.g., with elemental sulfur to yield the corresponding imidazole-2-thiones). Nevertheless, the actual presence of NHC in N,N'-disubstituted imidazolium acetate is still controversial. Moreover, theoretical studies seem to rule out the presence of NHC in such a polar environment as an IL. Aim of this Mini Review is to give the reader an up-to-date overview on the actual or potential presence of NHC in such an “organocatalytic ionic liquid,” both from the experimental and theoretical point of view, without the intent to be exhaustive on N,N'-disubstituted imidazolium acetate applications.

## Introduction

Ionic liquids (ILs), salts constituted of a large organic cation and an organic or inorganic anion not coordinated (usually liquid below 100°C), are gaining more and more popularity in many fields of Chemistry (Handy, 2011; Vekariya, 2017; Watanabe et al., 2017). Due to their physico-chemical properties their use is advantageous in view of a “greener” way of thinking Chemistry (Mohammad and Inammudin, 2012; Feroci et al., 2013a) although there are not sufficient studies on their possible toxicity (Ostadjoo et al., 2018). In particular, their high solvation ability, their virtually null vapor pressure, the relative easiness in removing them from the reaction mixture and recycle them spurred chemists to revisit established chemical procedures using them both as solvents and as reagents (Qureshi et al., 2014; Hajipour and Rafiee, 2015). Imidazolium ionic liquids are a class of ILs very often used in organic chemistry, as solvents and as precursors of N-heterocyclic carbenes (NHC), very efficient ligands and organocatalysts (Enders et al., 2007; Biju, 2019). In fact, the deprotonation of the C2-H in between the two imidazolium nitrogen atoms leads to the formation of a singlet carbene, which can act as a base and/or nucleophile. This deprotonation is usually carried out using strong bases (Sowmiah et al., 2009; Chiarotto et al., 2014) or by cathodic reduction (Gorodetsky et al., 2004; Feroci et al., 2016a), but it is known from decades (Olofson et al., 1964) that C2-H/D exchange can be induced by weaker bases, like Et_3_N. This led to the awareness of the possibility to incorporate a weak base into the imidazolium salt structure, i.e., as counter-ion, in order to have in the same reagent the acid (imidazolium cation, precursor of carbene) and the base (acetate ion). Imidazolium acetates are currently used in many fields of organic chemistry, but the actual presence of NHC into such ionic liquids is still debated: is an imidazolium acetate a mixture of IL and NHC, or the basicity of the anion (with respect to the acidity of the imidazolium cation) is not strong enough for this deprotonation in such a polar environment as the ionic liquid? This question has been faced from both theoretical and experimental point of view.

## Theoretical studies

**Scheme 1:**
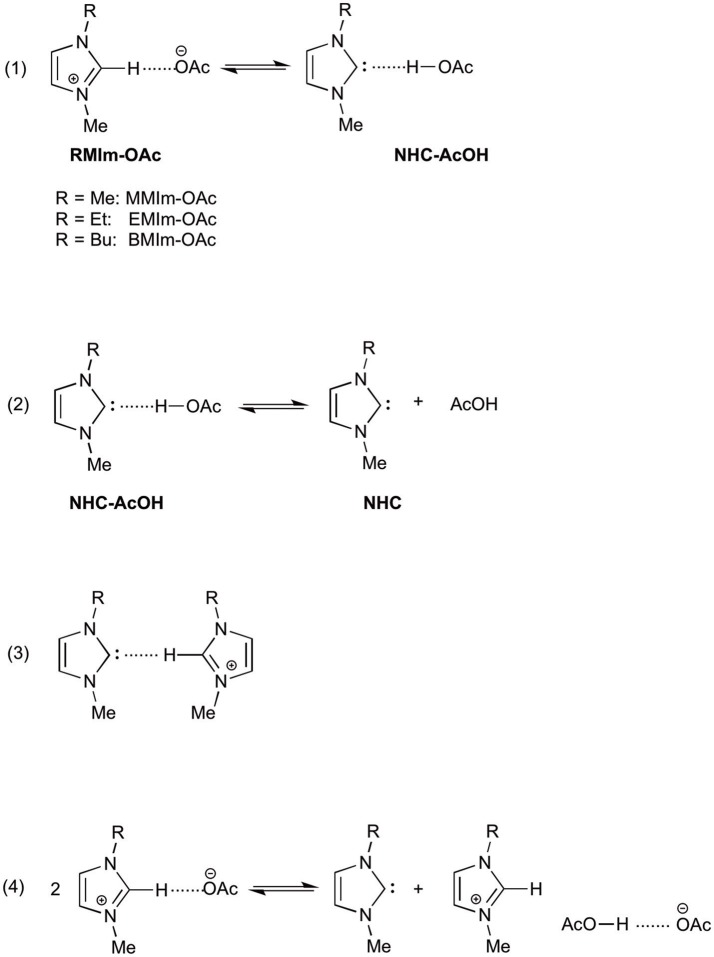
Possible structures and equilibrium reactions present in imidazolium acetate ionic liquids.

1-Ethyl-3-methylimidazolium acetate (EMIm-OAc) and 1-butyl-3-methylimidazolium acetate (BMIm-OAc) are among the most studied ILs potentially containing NHC, both theoretically and experimentally. The main question about the possibility of endogenous NHC in this kind of ILs arises from the very different pK_a_ of the N,N'-dialkylimidazolium cation and of acetic acid (about 22 and 12.3 in DMSO, respectively), which seems to rule out the possibility of a deprotonation of the imidazolium cation by acetate ion (although the pK_a_s in ILs are not known), leading to the formation of the corresponding NHC and acetic acid. To gain insights into the possibility of this proton transfer, Nyulászi and coworkers as early as 2010 studied computationally the system EMIm-OAc (at the B3LYP/6-31+G^*^ level), starting from the premise that the imidazolium cation is a good hydrogen-bond donor, while acetate anion is a hydrogen-bond acceptor and thus this ion pair could be represented as hydrogen bonded in the gas phase (Hollóczki et al., 2010). The authors found that the relative energy of the two hydrogen bonded structures (RMIm-OAc and NHC-AcOH, Scheme 1, equation 1) are almost identical and the barrier for their isomerization is low (3.6 kcal mol^−1^), rendering possible the existence of both isomers in the gas phase.

Qian et al. carried out quantum mechanical calculations to determine the reasons for the higher ability of imidazolium acetates over imidazolium chlorides in dissolving cellulose (Du and Qian, 2011). In this case also the effect of the solvent was evaluated. The authors calculated the free energy for the deprotonation reaction in the gas phase (−74.2 kcal mol^−1^), while in the MMIm-OAc ionic liquid the same calculation gave a value of +25.4 kcal mol^−1^, suggesting that the deprotonation process is favorable in the gas phase, but unfavorable in IL (in fact, the reactant are charged while the products are neutral and a polar solvent stabilizes more charged species). Furthermore, *ab initio* calculations allowed the authors to suggest a reaction mechanism in which the NHC-AcOH hydrogen bonded complex dissociates into NHC and AcOH (Scheme 1, equation 2), rendering NHC free to react with cellulose.

Later, Hollóczki and Kirchner (Thomas et al., 2014) reconsider the system EMIm-OAc taking into account the NHC solvation in such an ionic liquid. Starting from previous experimental data, the authors investigated the possible hydrogen bond between NHC and the EMIm^+^ cation (Scheme 1, equation 3) and its stabilizing effect. Unexpectedly, the authors found that such an interaction was not present in the studied system, as the C2-H site of the imidazolium cation was involved exclusively in an interaction with the acetate ion. This lack of stabilization is partially balanced out by a hydrogen bond between the carbene center and the alkyl substituent of the cation. The authors conclude that in a system like EMIm-OAc a latent carbene content can be observed, but due to the absence of a stabilizing effect by hydrogen bond between NHC and EMIm^+^ cation, the proton transfer between C2-H and AcO^−^ could be suppressed.

More recently (Gehrke and Hollóczki, 2017) Hollóczki and Gehrke questioned the actual presence of a NHC in an IL in which a weak base is present (starting from the high difference in pK_a_), suggesting instead a concerted mechanism for the imidazolium cation deprotonation and reaction with the substrate to yield the organocatalysed reaction products.

## Experimental studies

Due to the experimental evidence that imidazolium acetate ionic liquids act as organocatalysts in organocatalytic reactions (e.g., the benzoin condensation), many attempts were made in order to prove the presence of NHC in such an ionic liquid. In fact, the presence of NHC and acetic acid in the gas phase (due to the evaporation under vacuum of EMIm-OAc) was proved by photoelectron spectroscopy and mass spectroscopy (Hollóczki et al., 2010), but in the liquid phase only an indirect evidence of NHC presence was obtained, as benzoin was isolated by reaction of EMIm-OAc and benzaldehyde (Kelemen et al., 2011). This result spurred the authors to refer to EMIm-OAc as an “organocatalytic ionic liquid.” Almost at the same time, Rogers and coworkers (Rodríguez et al., 2011) confirmed the possibility to obtain NHC derived products (imidazole-2-thiones) by reaction of EMIm-OAc with elemental chalcogens, and suggesting for such an ionic liquid the name “proto-carbene.” Moreover, the same authors studied the effect of the addition of a proton donor (acetic acid, water) on this reaction and proposed a mechanism for the stabilization in IL of the neutral products derived from the deprotonation of EMIm^+^ cation by acetate anion. Such a mechanism (Scheme 1, equation 4) describes the formation of a hydrogen bonded dimer between acetic acid and acetate ion. On the other side, Welton and coworkers (Clough et al., 2013) carried out a study on the thermal degradation of EMIm-OAc with the identification of the neutral products obtained at high temperature (by thermogravimetric analysis coupled with mass spectrometry) and found that the main products derived from dealkylation reactions, while NHC formation was occurring to a small extent.

Kar and Sander (2015) demonstrated the reversibility of the imidazolium deprotonation reaction by acetate anion (Scheme 1, equation 1), carrying out experiments at very low temperature (9 K), on gas and condensed phases, monitoring the system by IR spectroscopy. They found that for EMIm-OAc both ionic liquid and neutral species (NHC and AcOH) coexist at very low temperatures and if the vapor phase is condensed at 9 K, only NHC and acetic acid are present (and no ionic species). Moreover, if the temperature is increased to room temperature, a proton transfer is active, leading again to the ionic liquid EMIm-OAc.

The demonstration of the potential presence of NHC in imidazolium acetates was obtained by many groups, carrying out successfully catalyzed or stoichiometric reactions (Lambert et al., 2016; Baumruck et al., 2017; Binks et al., 2018; Pandolfi et al., 2018), but in no case any attempt to evidence the actual presence of NHC was done. This last topic was faced in a few papers. Inesi and coworkers (Chiarotto et al., 2015, 2017) studied this question by cyclic voltammetry, starting from the fact that NHC is an electroactive species and it can be oxidized at the anode at a potential around +0.5 V (vs. Ag), in pure imidazolium acetate ionic liquid. This investigation was carried out registering the cyclic voltammetries of pure IL at different temperatures and comparing the electrochemical behavior of BMIm-OAc with that of an imidazolium ionic liquid containing a less basic anion, BMIm-Cl. The authors found that at a temperature higher of 100°C the cyclic voltammetry of BMIm-OAc showed the presence of the corresponding carbene, while starting from the corresponding chloride ionic liquid, the oxidation peak of NHC was not present in 25–150°C interval of temperatures. The authors thus infer that the acetate anion is a base strong enough to deprotonate C2-H of the BMIm^+^ cation and that this deprotonation reaction at temperatures higher than 100°C leads to the formation of a detectable amount of NHC. Suzer and coworkers (Gokturk et al., 2017) recently confirmed by XPS analysis the attribution of the anodic peak around +0.5 V to the oxidation of NHC, electrogenerated by cathodic reduction. In fact, imidazolium NHCs can also be generated by cathodic cleavage of the C2-H bond to yield NHC and molecular hydrogen (Gorodetsky et al., 2004; Feroci et al., 2016b,c).

Welton and coworkers (Daud et al., 2017) consider that all the observations reported in the literature about the presence of NHC in imidazolium acetate ionic liquids do not confirm the actual presence of carbenes in ILs, but just indicate their accessibility and that in order to have NHC derived products it is necessary the presence of a “NHC trap,” i.e., a reagent or the electrode. Moreover, they believe that a concerted mechanism to NHC derived products can always be claimed in alternative to a multistep one (with the formation of NHC as a distinct molecule). In order to gain evidences on the actual or potential presence of NHC in EMIm-OAc, a kinetic study was carried out on a possible deuterium isotope effect in the formation of the adduct between NHC and an aromatic aldehyde (Breslow intermediate) starting from EMIm-OAc with a C2-D or C2-H group. The absence of a deuterium isotope effect allowed the authors to exclude a concerted mechanism and to confirm the actual presence of NHC in EMIm-OAc.

Apart from acetates (and other imidazolium organic carboxylates), other imidazolium ionic liquids containing basic anions are reported to be useful reagents for NHC derived reactions. Among them hydrogen carbonate and hydroxide are particularly important. As regards 1,3-dialkylimidazolium hydrogen carbonates, it is reported that these ionic liquids are in equilibrium with the corresponding NHC-CO_2_ adducts (vide infra), demonstrating the possibility to afford NHCs (see for example: Fèvre et al., 2012; Zhao et al., 2017) by releasing of carbon dioxide due to high temperature effect or, at room temperature, using a solvent favoring the carbene generation (as THF or toluene).

As for 1,3-dialkylimidazolium hydroxides, it is a different story. In fact, although hydroxide anion is a noteworthy basic species (or precisely for this reason), imidazolium hydroxides are very rarely used as organocatalysts or reagents (see for example: Rajesh et al., 2012), due to their instability. In fact, the reaction between 1,3-dialkylimidazolium cation and hydroxide anion leads mainly to ring opening products (Yuen et al., 2013; Long and Pivovar, 2014), rendering in most of cases unuseful this class of imidazolium ionic liquids (although the ring opening seems to be directly related to NHC formation, Hollóczki et al., 2011).

The literature survey on imidazolium ionic liquids containing basic anions other than acetate is not intended to be complete.

## The presence of CO_2_

**Scheme 2:**
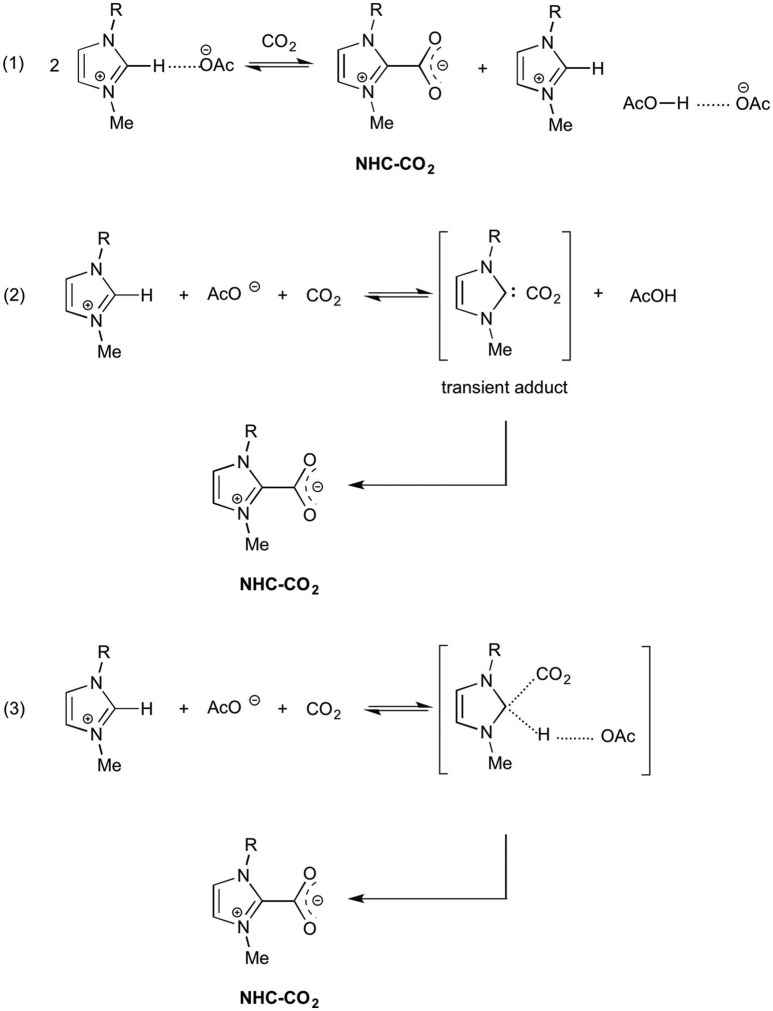
Possible reactions in imidazolium acetate IL-CO_2_ mixtures.

Besides the various applications in organic chemistry, electrochemistry or material chemistry, ionic liquids gained popularity in the field of carbon dioxide capture and storage (CCS, e.g., in CO_2_ absorption from industrial waste gases), as carbon dioxide catch and release reagents (de Robillard et al., 2013; Feroci et al., 2013b). Physisorption is the mechanism for such a CO_2_ capture in ILs, but chemisorption was invoked for some functionalized ILs. In particular, the solubility of carbon dioxide in imidazolium acetate ionic liquids is remarkable and this very high solubility was charged to chemisorption. In order to fully understand the nature of such an interaction (with acetate anion, imidazolium cation or NHC), many theoretical and experimental studies have been carried out and such papers shed light on the presence of endogenous NHC in imidazolium acetate ILs. Rogers and coworkers (Gurau et al., 2011) reported the single-crystal X-ray structures of EMIm-OAc and EMIm-OAc-CO_2_ mixture and found that no NHC was present when studying pure IL (as expected for the solid phase). On the contrary, after CO_2_ bubbling the corresponding NHC-CO_2_ adduct was present, along with the AcO^−^/AcOH dimer (Scheme 2, equation 1), demonstrating the formation of NHC and suggesting a two-step mechanism (formation of NHC and then reaction with CO_2_ to give the adduct).

Besnard and coworkers (Besnard et al., 2012; Cabaço et al., 2012) carried out theoretical (DFT) and experimental (Raman, NMR) studies to understand the high CO_2_ solubility in BMIm-OAc and suggested a two-step mechanism dominated by an irreversible chemical reaction leading to the formation of the NHC-CO_2_ adduct (Scheme 2, equation 2). In the suggested mechanism a first reaction leads to the formation of a transient NHC, stabilized by the interaction with carbon dioxide; although the NHC formation is not favored in the liquid phase, it is triggered by the interaction with CO_2_. The transient intermediate then isomerizes to NHC-CO_2_ adduct. Moreover, the acetic acid molecule produced in this reaction interacts with the acetate anion to give a hydrogen bonded dimer.

Kirchner and Hollóczki (Hollóczki et al., 2013a,b) studied the EMIm-OAc-CO_2_ system by AIMD simulations and static quantum chemical calculations and found that in the liquid phase the NHC formation is facilitated by the physically absorbed carbon dioxide, leading to the chemically absorbed CO_2_ (NHC-CO_2_ adduct). Moreover, the authors underline the fact that the occurrence of NHC induced reactions in EMIm-OAc does not prove that NHC is present in this IL, but only that it is accessible.

DFT and ONIOM calculations on imidazolium acetate-CO_2_ system, both in the gas phase and in the liquid phase, considering the one-step and the two step mechanisms (Scheme 2, equations 1 and 2) allowed Damodaran and coworkers (Mao et al., 2016) to propose a new two-step reaction mechanism: at first the addition reaction between the imidazolium cation (at C2) and carbon dioxide, then the deprotonation of the C2-H of the adduct to yield NHC-CO_2_. The authors emphasize the important role played by IL in the stabilization of the products.

In a recent paper, (Yan et al., 2017) reporting *ab initio* results on EMIm-OAc, the effect of solvation is underlined also by Kim and coworkers. The authors exclude the presence of NHC in such an ionic liquid and suggest that acetate anion can deprotonate the imidazolium cation only in a non-polar or extremely weakly polar environment (solvent). Moreover they suggest that the reaction of EMIm-OAc with carbon dioxide to yield NHC-CO_2_ adduct is a concerted process (Scheme 2, equation 3), in which the intermediate has a sp^3^ C2 which subsequently evolves toward the NHC-CO_2_ adduct.

These NHC-CO_2_ adducts are also useful NHC masked organocatalysts for the carboxylation of organic compounds (see as examples: Tommasi and Sorrentino, 2006; Desens and Werner, 2016; Stewart et al., 2016) and NHC-transfer agent in the synthesis of NHC-metal complexes (see as examples: Voutchkova et al., 2007; Voutchkova and Crabtree, 2010; Li et al., 2011).

## Conclusions

The overview of the last decade literature on the possible presence of NHC in imidazolium acetate ionic liquids evidences that the question of the actual or potential presence of NHC in such ionic liquids is still debated. On one side, theoretical calculations seem to exclude the possibility of NHC presence in imidazolium acetate, due to the highly polar environment which should strongly unfavor the proton transfer from imidazolium C2-H to acetate anion, and thus transform charged species into neutral molecules. On the other side, experimental studies demonstrate the possibility of using imidazolium acetates as a reservoir of NHC, giving rise to the formation of products in which it is necessary the formation of catalytic or stoichiometric amounts of carbene. It is still possible that the deprotonation equilibrium at room temperature lies far toward the charged species and the presence of a “carbene-trap” (always present in NHC-induced organic reactions) or an irreversible subsequent reaction is necessary in order to move such an equilibrium toward the formation of NHC and acetic acid. Moreover, it is possible that in some cases the reactions between NHC and its trap and NHC formation are concerted (as theorized for the reaction between NHC and CO_2_), while in others they are a two-step process (as demonstrated in the Breslow intermediate formation). The present literature data exclude the possibility to isolate or even evidence the presence of NHC in imidazolium acetates (either for its presence as a strongly hydrogen bonded adduct, or for its very low concentration), but it is not to be excluded that in the future it would be possible to isolate from these systems stable NHCs (like Arduengo's ones).

Anyway, it is undeniable that imidazolium acetate ILs are “organocatalytic ionic liquids” or “proto-carbenes” and that their reactivity is so peculiar that their use is highly suggested in many fields of Chemistry.

## Author contributions

All authors listed have made a substantial, direct, and intellectual contribution to the work, and approved it for publication.

### Conflict of interest statement

The authors declare that the research was conducted in the absence of any commercial or financial relationships that could be construed as a potential conflict of interest.
